# A complicated case of osteodiscitis and retroperitoneal abscesses in an immunosuppressed patient

**DOI:** 10.1186/1471-2334-14-S7-P2

**Published:** 2014-10-15

**Authors:** Alexandra Elena Duca, Octavia Dincă, Caterina Livia Pahomi-Nicolae, Alina-Cristina Neguț, Anca Streinu-Cercel, Oana Săndulescu, Bogdan Haineala, Silviu Margaritis, Marian Munteanu, Adina Elena Ilie, Monica Andreea Soica, Adrian Streinu-Cercel

**Affiliations:** 1 National Institute for Infectious Diseases "Prof. Dr. Matei Balş", Bucharest, Romania; 2 Carol Davila University of Medicine and Pharmacy, Bucharest, Romania; 3 Fundeni Clinical Institute, Bucharest, Romania; 4 Central Universitary Emergency Military Hospital Dr Carol Davila, Bucharest, Romania

## Background

Frequent pathogens responsible for osteodiscitis include *Staphylococcus aureus*, enteric Gram-negative bacilli and *Mycobacterium tuberculosis*.

## Case report

A 59 year-old female with type-2 insulin-dependent diabetes presented to our clinic in June 2014 for fever, productive cough and left chest pain. On admission, the clinical exam revealed lower left lung absent breath sounds, right mastectomy and intensely impaired mobility. The laboratory reports showed leukocytosis and thrombocytosis. The CT scan and the thoracolumbar spine MRI exam described T12-L2 osteodiscitis with osteodiscal abscess, two massive right and left retroperitoneal abscesses close to both ureters, and left pleural effusion as an extension of the left abscess.

Her medical history revealed that in June 2013 she had suffered a traumatic comminuted L1 fracture, with long-term lumbar pain that progressed to left hip and thigh pain. She also had a history of surgery and radio-chemotherapy for breast cancer in 2011. As the pathogenic agent had not been isolated yet, the patient was started on ertapenem, linezolid and anti-tuberculosis (antiTB) therapy. She was transferred to neurosurgery, where a T12 hemilaminectomy was performed, leading to improved mobility. Cultures identified *Serratia marcescens*, and smears showed frequent polymorphonuclear cells; therapy was changed to tigecycline, and antiTB treatment was stopped.

As the size of abscesses increased on serial ultrasound scans 2 weeks apart, the patient was transferred to urology, where the abscesses were drained and a drainage tube was placed. The abscess cultures came back negative, but the smear showed frequent polymorphonuclear cells. The patient was switched to oral ciprofloxacin and rifampin, with clinically favorable evolution. After 2 weeks of oral therapy she displayed no leukocytosis, no biological inflammatory syndrome and an ultrasound exam showed partially drained abscesses. We performed smears and cultures from the drainage tube fluid, for all suspected pathogens, including *Mycobacterium tuberculosis*. The smears showed Gram-positive cocci and 15% polymorphonuclear cells and 45% lymphocytes. The Ziehl-Neelsen stain was negative. All cultures are still in progress. The patient is scheduled for urologic evaluation and a CT scan, to decide whether open surgery is required.

## Conclusion

*Serratia marcescens* can be considered as pathogenic agent in immunosuppressed patients. However, *M. tuberculosis* cannot be ruled out in this case, given the insidious evolution and the size of abscesses, particularly as the patient displayed favorable evolution under ciprofloxacin and rifampin. In difficult to treat cases, antimicrobial treatment may become limited and a multidisciplinary approach is needed to ensure the best management.

## Consent

Written informed consent was obtained from the patient for publication of this Case report and any accompanying images. A copy of the written consent is available for review by the Editor of this journal.

